# Design and preparation of nanoarchitectonics of LDH/polymer composite with particular morphology as catalyst for green synthesis of imidazole derivatives

**DOI:** 10.1038/s41598-022-15582-z

**Published:** 2022-07-04

**Authors:** Nastaran Ghanbari, Hossein Ghafuri

**Affiliations:** Catalysts and Organic Synthesis Research Laboratory, Department of Chemistry, Iran University of Science and Technology, Tehran, 16846‑13114 Iran

**Keywords:** Medical research, Chemistry

## Abstract

This paper was designed and prepared a new nanoarchitectonics of LDH/polymer composite with specific morphology. For this purpose, CTAB surfactant was used to control the morphology of layered double hydroxide (LDH) and to prepare LDH/polymer nanocomposites (LDH–APS–PEI–DTPA). The polymer was synthesized using diethylenetriaminepentaacetic acid (DTPA), polyethylenimine and used with LDH to form a nanocomposite with high thermal stability. Subsequently, the prepared nanocomposite was identified using FTIR, EDX, TGA, XRD, FESEM, and BET techniques. In addition, the prepared LDH–APS–PEI–DTPA nanocomposite was used as a heterogeneous and recyclable catalyst for the synthesis of imidazole derivatives under green conditions. The results showed that the LDH–APS–PEI–DTPA nanocomposite benefit from suitable morphology, simple preparation, high catalytic activity, and high surface area. Also, the proposed LDH–APS–PEI–DTPA heterogeneous catalyst showed high stability and reusability for five consecutive runs which was consistent with the principles of green chemistry.

## Introduction

Over the past decade, extensive research has focused on polymer nanocomposites which are composed of a polymer matrix with dispersed nanoscale reinforcing particles^1–4^. Generally, nanocomposites show much better mechanical properties than similar micro-sized composites^5–9^. Classical composite theory predicts that improved bonding between the polymer matrix and the other components leads to the improved mechanical properties^8^. The composition of layered inorganic fillers within polymer matrices for the formation of polymer/layered inorganic nanocomposites is of great importance due to their distinctive properties^6,10–12^. In this regard, layered double hydroxide (LDH)/polymer nanocomposites belong to an important class of polymer/layered inorganic nanocomposites because they have significantly improved thermal stability and physical properties^13–19^. LDH has adjustable sheet-like structure and can be synthesized by the following methods: urea hydrolysis, co-precipitation, hydrothermal synthesis, simultaneous precipitation, and ion exchange^20–23^. LDH laminates are composed of metal cations and hydroxides, in which anions are placed between the layers, i.e. interlayer ions^24^. Therefore, laminate ions, valence states, and interlayer anions in the LDH are adjustable, making LDH a promising candidate for various applications^25^. Also, due to its unique layered structure, it can increase the specific surface area and active sites in the composites so that it can increase the possibility of mixing with most of materials^26^. Various LDH catalysts based on metals such as Ti, Fe, Mg, Ni, Cu, etc., have been used in the catalytic reaction^16,27–30^.

The chemical structure of polyethylenimine (PEI) is composed of ethylene imine (aziridine) or oxazoline monomers, which result in branched or linear polymeric backbones, respectively. This molecule is a simple replicate of the CH_2_–CH_2_–NH ethylene imine motif. PEI is a water-soluble branched cationic polymer that has several active amine groups in its branched chains^31^.

Diethylenetriaminepentaacetic acid (DTPA) or pentetic acid is an aminopolycarboxylic acid consisting of a diethylenetriamine with five carboxymethyl groups. It is a white solid with limited solubility in water^32^. The molecule can be viewed as an expanded version of EDTA and is used similarly^33^.

Also, among heterocyclic compounds imidazole derivatives have attracted special attention due to their biological and medicinal properties^34,35^. This group of 1,3-diazoles exhibits therapeutic behaviors such as antibiotics and antifungals. Imidazole compounds are used as a medicinal nucleus in some drugs such as cimetidine, ketoconazole, daclatasvir^36^ and nitroimidazole, which is an antibiotic for the treatment of gastrointestinal infections. In the recent decades, the synthesis of imidazole derivatives in the presence of various catalysts has been reported. Homogeneous or heterogeneous catalysts reported for the synthesis of imidazole derivatives include molecular iodine^37^, molecularly imprinted polymer^38^, p-toluenesulfonic acid^39^, graphene oxide-chitosan composite^40^, etc. Imidazole derivatives despite their advantages have disadvantages due to the use of toxic solvents, high loading of the catalyst, low production efficiency, and the cost of metal catalysts.

There have been numerous reports of the use of LDHs in multicomponent reactions (MCRs)^30,41,42^. In several cases it was found that a change in the calcination process conditions leading to the formation of mixed oxides or a change in the ratio of metal cations is a useful parameter for a highly selective reaction^43,44^. Examples of these reactions include Biginelli reaction^45^, Hantzsch reaction^46^, choromen reaction^47^, and so on. The LDH–APS–PEI–DTPA (**1**) nanocomposite prepared here has many advantages over previously reported work such as short reaction time, high efficiency, easy separation in multi-component reactions.

**Figure 1:**
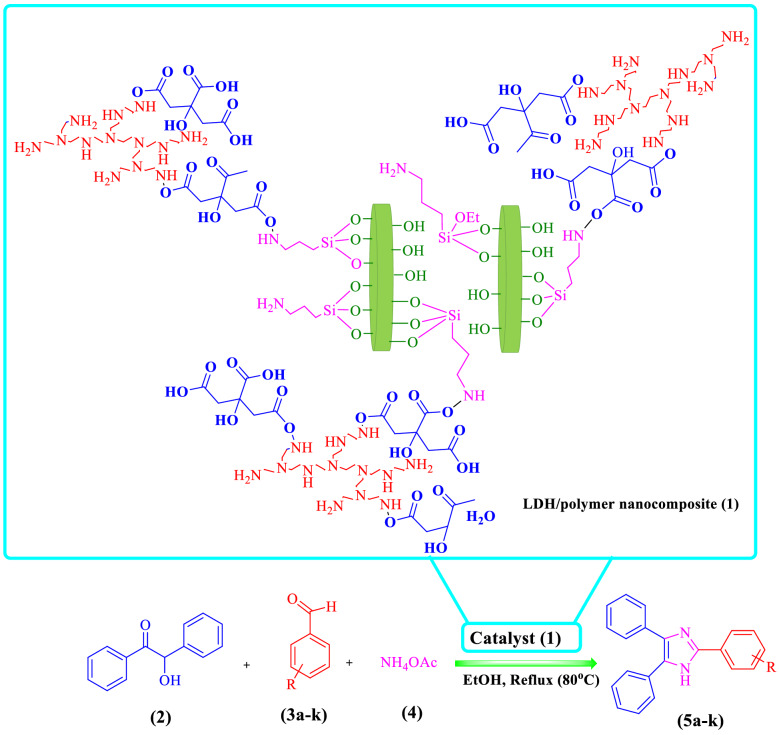
LDH–APS–PEI–DTPA nanocomposite (**1**)-catalyzed synthesis of imidazole derivatives through a multicomponent reaction of benzoin (**2**), aldehyde derivatives (**3a–k**), and ammonium acetate (**4**) in EtOH under reflux conditions.

In this study, a new nanoarchitectonics of LDH–APS–PEI–DTPA (**1**) composite designing and prepared using Mg–Al LDH with a new morphology and a new polymer was prepared. Also, the prepared LDH–APS–PEI–DTPA nanocomposite (**1**) was used as a highly efficient and recoverable heterogeneous catalyst for facile one-pot green synthesis of imidazole derivatives via three-component addition of benzoin (**2**), aldehydes (**3a**–**k**), and ammonium acetate (**4**) (Fig. 1).

## Results and discussion

**Figure 2:**
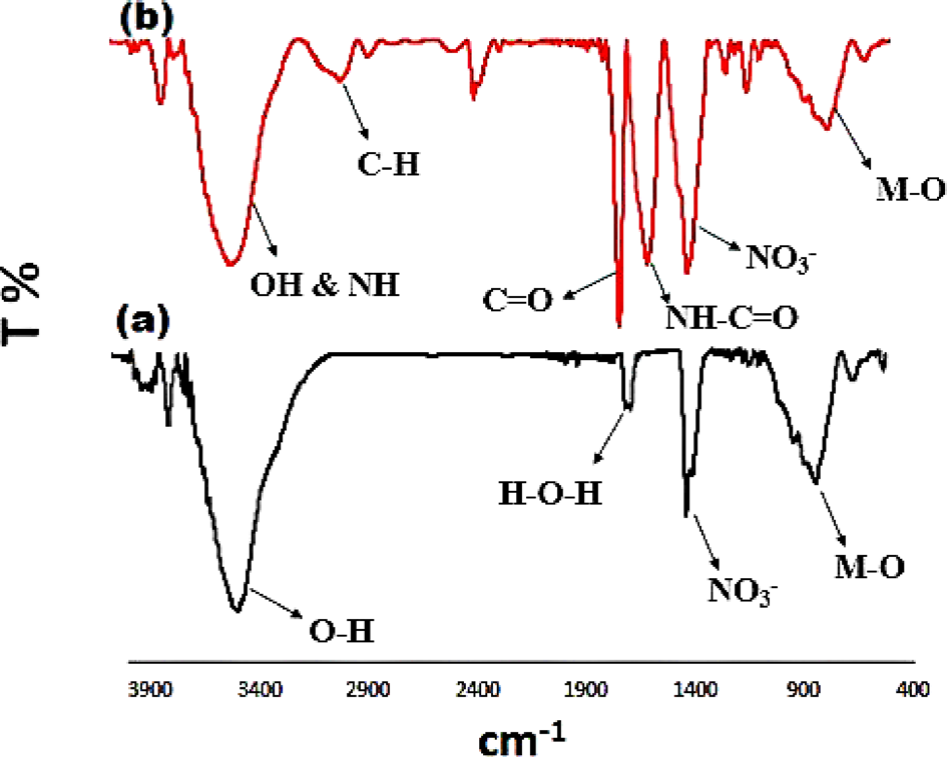
FTIR spectra of Mg–Al LDH (**a**) and LDH–APS–PEI–DTPA nanocomposite (**b**).

Figure 2 shows the FTIR spectra of Mg–Al LDH (**2a**) and LDH–APS–PEI–DTPA nanocomposite (**1**, **2b**). Figure 2a shows the FTIR spectra of Mg–Al LDH which has broad adsorption band at around 3430 cm^−1^ for O–H groups. The absorption band at 1624 cm^−1^ is attributed to the H–O–H bending vibration of the interlayer water. In addition, the absorption band at 1370 cm^−1^ is related to nitrate anions. The absorption band at 882–584 cm^−1^ corresponds to the Al–OH and M–O bonds, where M can be Mg or Al. Therefore, the observed absorption bands confirm that the change in morphology of Mg–Al LDH did not lead to a change in its chemical structure.

Figure 2b shows the FTIR spectra of LDH–APS–PEI–DTPA nanocomposite (**1**). The absorption band at 3466 cm^−1^ is related to N–H groups. Also, the absorption band at around 2970 cm^−1^ corresponds to C-H aliphatic. Furthermore, the absorption bands at 1710 cm^−1^ and 1654 cm^−1^ belong to the stretching vibrations of C=O bond of carboxylic acid and amide groups, respectively. In addition, the adsorption band at 1402 cm^−1^ is attributed to the stretching vibrations of nitrate anions. The absorption bands at 1234 cm^−1^, 1138 cm^−1^, and 800–500 cm^−1^ are related to Si–O–Si, C–O and M–O, respectively, where M can be Mg or Al.

**Figure 3:**
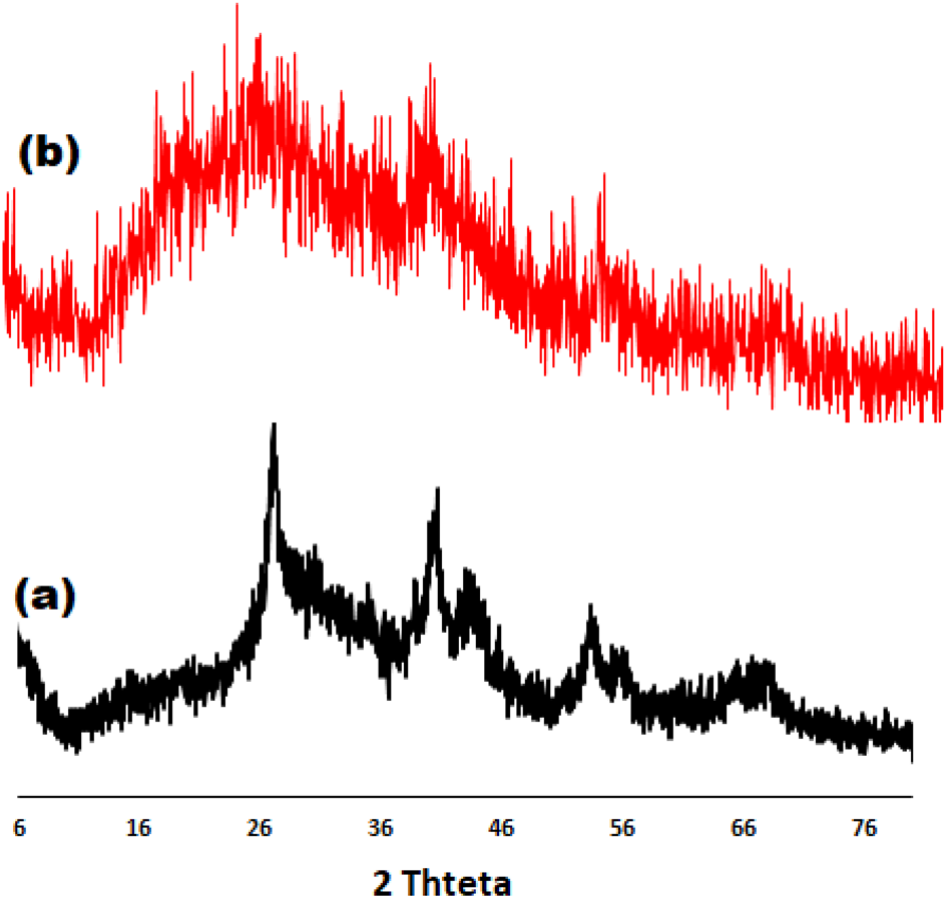
XRD patterns of Mg–Al LDH (**a**) and LDH–APS–PEI–DTPA nanocomposite (**b**).

Figure 3 shows XRD patterns of Mg–Al LDH (**2a**) and LDH–APS–PEI–DTPA nanocomposite (**1**, **3b**). The XRD pattern of Mg–Al LDH shows sharp and symmetrical reflections at 2θ of 26.11°, 30.93°, 34.88°, 40.61°, 43.40°, 53.32°, 63.09°and 66.31°, respectively, which are characteristic of the Mg–Al LDH structure. Thus, the peaks seen in the XRD pattern show that the morphological change of Mg–Al LDH did not lead to a change in its chemical structure (Fig. 3a). Also, Fig. 3b shows the XRD pattern of the LDH–APS–PEI–DTPA nanocomposite (**1**), which confirms the presence of LDH and polymer compounds in the composite structure. There is a relatively sharp peak at 2θ = 20°–30°, which indicates the amorphous structure of the polymer. Also, Fig. 3b shows sharp and symmetrical reflections at 2θ of 26.14°, 35.90°, 41.11°, 44.20°, 54.31° and 67.29°, respectively, which are characteristic of the LDH–APS–PEI–DTPA nanocomposite (**1**).

**Figure 4:**
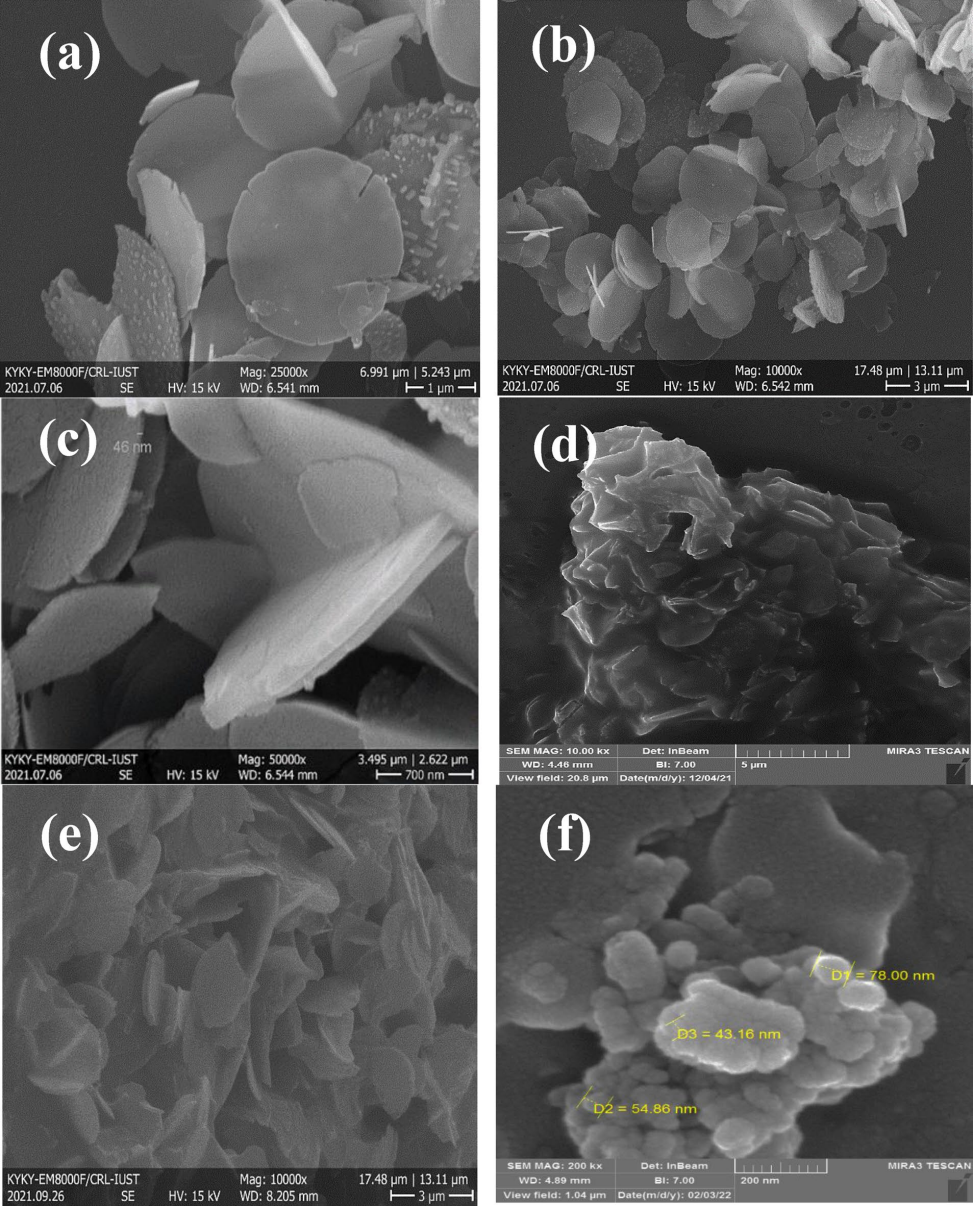
FESEM images of the Mg–Al LDH (**a**,**b**,**c**), and LDH–APS–PEI–DTPA nanocomposite (**1**,**d**,**e**,**f**).

Figure 4 shows the FESEM images of Mg–Al LDH (**a**, **b**, **c**) and LDH–APS–PEI–DTPA nanocomposite (**1**, **d**, **e**, **f**). FESEM images of pristine Mg–Al LDH with novel morphology showing stacked circular plate-like crystals with a diameter around 46 nm in (Fig. 4a–c). These images confirm the morphological change of Mg–Al LDH from hexagonal to circular plates. Also, Fig. 4d–f shows images of LDH–APS–PEI–DTPA nanocomposite (**1**). Interestingly, the plate diameter of LDH–APS–PEI–DTPA nanocomposite (**1**) increases and shows larger platelets with dimensions of 43 to 78 nm. Therefore, after combining LDH with polymer, the surface became irregular and the thickness of LDH layers was increasing.

**Figure 5:**
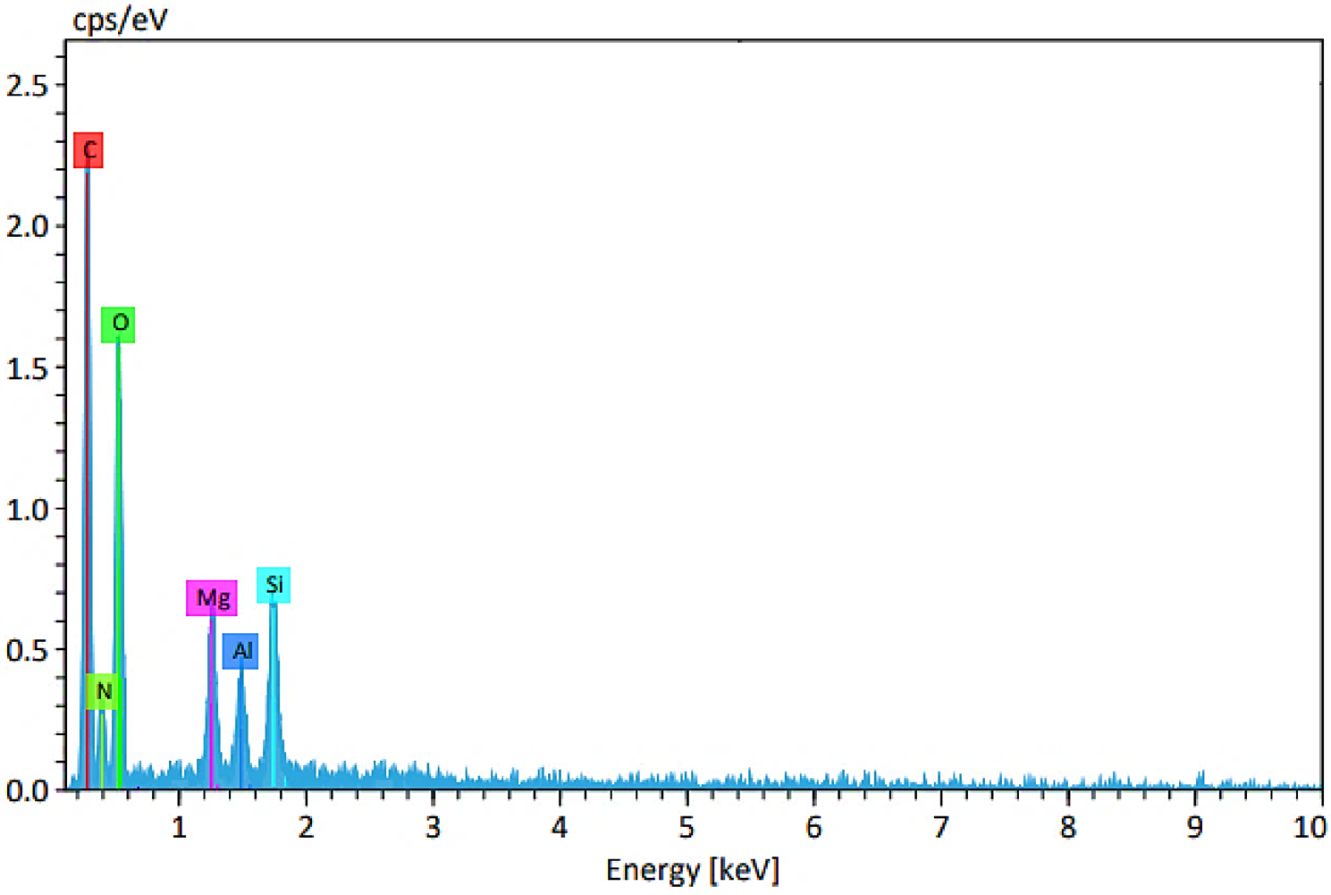
EDX spectra of LDH–APS–PEI–DTPA nanocomposite (**1**).

EDX analysis of LDH–APS–PEI–DTPA nanocomposite (**1**) shows the presence of C (36.31%), O 37.56%), N (13.88%), Al (5.43%), Mg (4.29%) and Si (2.53%) elements which confirm the structure of the prepared nanocomposite (Fig. 5).

**Figure 6:**
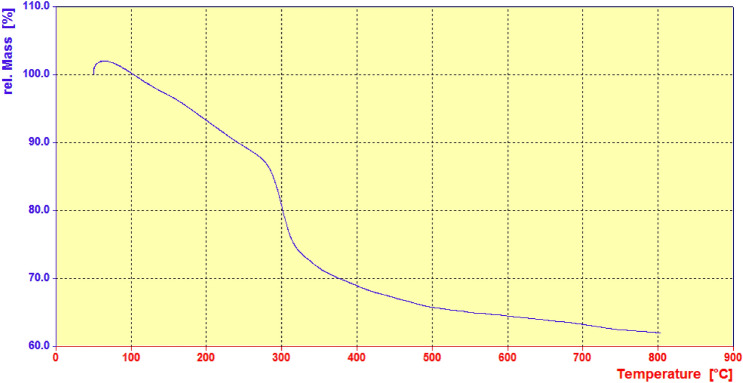
TGA curve of the LDH–APS–PEI–DTPA nanocomposite (**1**).

TGA analysis was also performed to evaluate the thermal stability of LDH–APS–PEI–DTPA nanocomposite (**1**) in the range of 50–800 °C. The TGA curve of LDH–APS–PEI–DTPA nanocomposite (**1**) in Fig. 6 shows three weight losses. The first weight loss at 50–100 °C is caused by the evaporation of surface water molecules and solvent adsorbed on the LDH–APS–PEI–DTPA nanocomposite (**1**), while and the second weight loss at 160–270 °C is attributed to the thermal decomposition of organic component in LDH–APS–PEI–DTPA nanocomposite (**1**). The third weight loss occurred in the range of 280–500 °C, which can be associated with the condensation of LDH. Therefore, the obtained results confirm the successful preparation of LDH–APS–PEI–DTPA nanocomposite (**1**).

**Figure 7:**
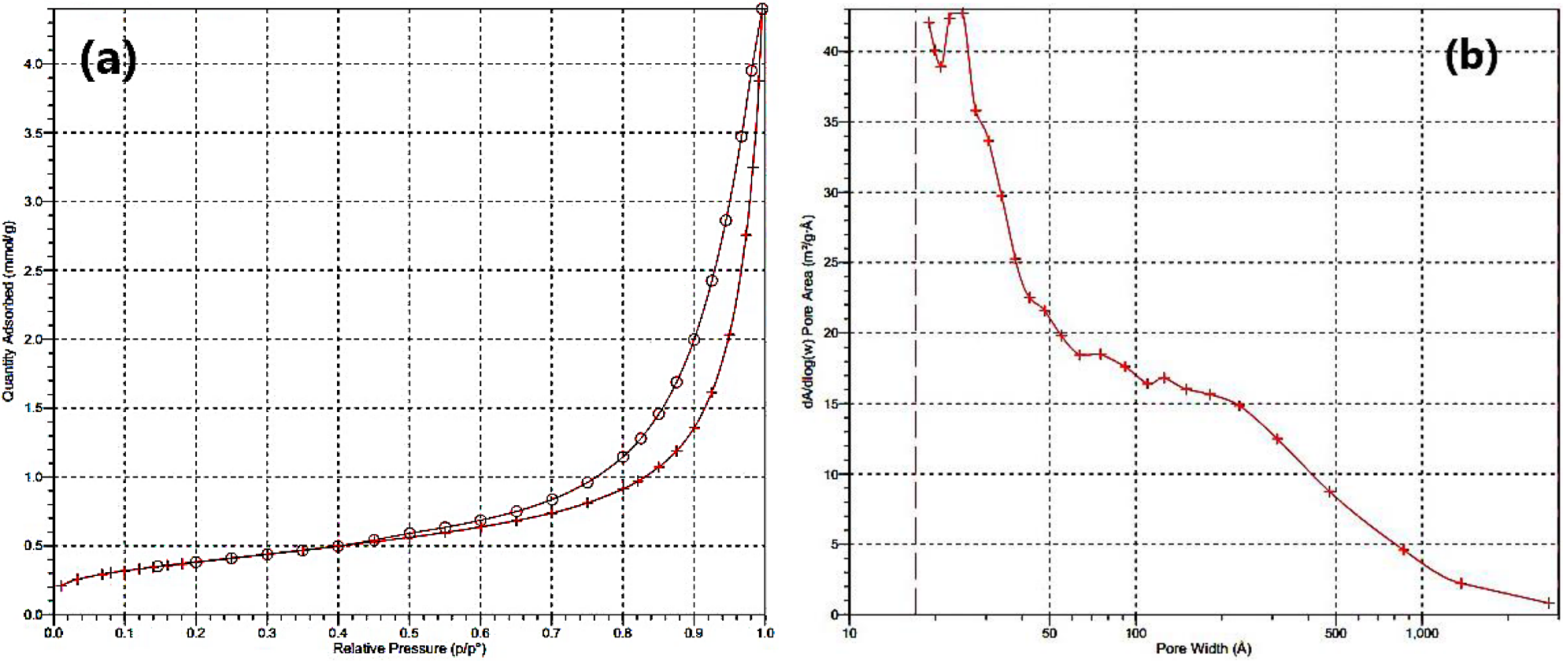
N_2_ adsorption–desorption curves for the LDH–APS–PEI–DTPA nanocomposite (**1**).

Figure 7 shows the N_2_ adsorption–desorption isotherms for LDH–APS–PEI–DTPA nanocomposite (**1**) structures. As shown, the surface area of the LDH–APS–PEI–DTPA nanocomposite (**1**) is about 160 m^2^ g^−1^ and the pore-size distribution is 0.09 cm^3^ g^−1^.

### Optimization of the reaction conditions using LDH–APS–PEI–DTPA nanocomposite (**1**) catalyst

In the following, LDH–APS–PEI–DTPA nanocomposite (**1**) was used for the synthesis of imidazole derivatives. Hence, the condensation was evaluated between benzoin (**2**, 1 mmol), aldehyde (**3**, 1 mmol), and ammonium acetate (**4**, 2.5 mmol) as the model reaction. First, the model reaction was investigated in the absence of catalyst using different solvents at different temperatures (Table 1, Entries 1–6). As shown in Table 2, in the absence of the catalyst, the desired product was not produced after 3 h. however, in the presence of 5 mg of LDH–APS–PEI–DTPA nanocomposite (**1**), the desired product 5a was formed with 40 to 85% production yield (Table 1, Entry 7–10). The desired product was formed in EtOH solvent at room temperature after 4 h with 40% yield (Table 1, entry 7). Also, the model reaction was carried out in EtOH at 50 °C for 4 h with 60% yield (Table 1, Entry 8). Next, the desired product was obtained in H_2_O under reflux conditions with a yield of 30% (Table 1, entry 9). Despite the negative performance of H_2_O in the model reaction, a yield of 85% was obtained in EtOH solvent under reflux conditions using 7 mg LDH–APS–PEI–DTPA nanocomposite (**1**) (Table 1, entry 10). According to the obtained results, EtOH solvent under reflux conditions was used as the optimal synthesis condition for the subsequent experiments. Also, to determine the optimal catalyst value, the model reaction was performed in EtOH solvent under reflux conditions in the presence of 3, 5, 7 and 10 mg of the synthesized catalyst (Table 1, entry 10–12). Therefore, 5 mg of the LDH–APS–PEI–DTPA nanocomposite (**1**) catalyst and EtOH solvent under reflux conditions were determined as the optimal conditions for the reaction.

**Table 1:** Synthesis of imidazole derivatives **5a**–**k** by the three-component condensation of benzoin (**2**), aldehyde derivatives **3a**–**k**, and ammonium acetate (**4**) in the presence of LDH–APS–PEI–DTPA nanocomposite (**1**).


Entry	Catalyst	Conditions	Catalyst loading (mg)	Time (min)	Yield (%)
1	–	Solvent-Free/RT	**–**	360	**–**
2	–	EtOH/RT	–	360	**–**
3	–	H_2_O/RT	–	360	Trace
4	–	EtOH/50 °C	–	360	Trace
5	–	EtOH/Reflux	–	360	20
6	–	H_2_O/Reflux	–	360	Trace
7	LDH–APS–PEI–DTPA nanocomposite (**1**)	EtOH/RT	5	360	35
8	LDH–APS–PEI–DTPA nanocomposite (**1**)	EtOH/50 °C	5	300	60
9	LDH–APS–PEI–DTPA nanocomposite (**1**)	H_2_O/Reflux	5	180	65
10	LDH–APS–PEI–DTPA nanocomposite (**1**)	EtOH/Reflux	7	60	85
11	LDH–APS–PEI–DTPA nanocomposite (**1**)	EtOH/Reflux	5	60	98
12	LDH–APS–PEI–DTPA nanocomposite (**1**)	EtOH/Reflux	3	60	75

Reaction conditions: Benzoin (**2**, 1 mmol), 4-chlorobenzaldehyde (**3b**, 1 mmol), ammonium acetate (**4**, 2.5 mmol), and LDH–APS–PEI–DTPA nanocomposite (**1**).

RT: room temperature.

**Table 2:** Synthesis of imidazole derivatives **5a**–**k** from three-component condensation of benzoin (**2**), aldehyde derivatives **3a**–**k**, and ammonium acetate (**4**) in the presence of LDH–APS–PEI–DTPA nanocomposite (**1**).


Entry	Product	Aldehyde	Time (min)	Yield (%)	Melting point (°C)	
					Observed	Reported
1			50	97	273–271	272–273^48^
2			60	98	261–263	260–262^49^
3			65	95	198–200	197–199^50^
4			65	95	176–178	177–178^50^
5			75	70	300–297	301–302^50^
6			90	70	200–201	200–202^51^
7			55	85	233–235	232–235^50^
8			55	90	227–229	227–228^50^
9			75	80	255–258	256–257^51^
10			60	90	250–252	250–251^51^
11			60	85	255–258	256–258^48^

Reaction conditions: Benzoin (**2**, 1 mmol), benzaldehyde (**3a**–**k**, 1 mmol), and ammonium acetate (**4**, 2.5 mmol) in the presence of 5 mg LDH–APS–PEI–DTPA nanocomposite (**1**) in EtOH under reflux conditions.

In order to extend the catalytic application of LDH–APS–PEI–DTPA nanocomposite (**1**), three-component condensation of benzoin (**2**), aldehyde derivatives (**3a**–**k**), and ammonium acetate (**4**) were performed under optimal conditions for the synthesis of imidazole derivatives. The results are summarized in Table 2.

### The proposed mechanism for the synthesis of imidazole derivatives in the presence of LDH–APS–PEI–DTPA nanocomposite (**1**)

**Figure 8:**
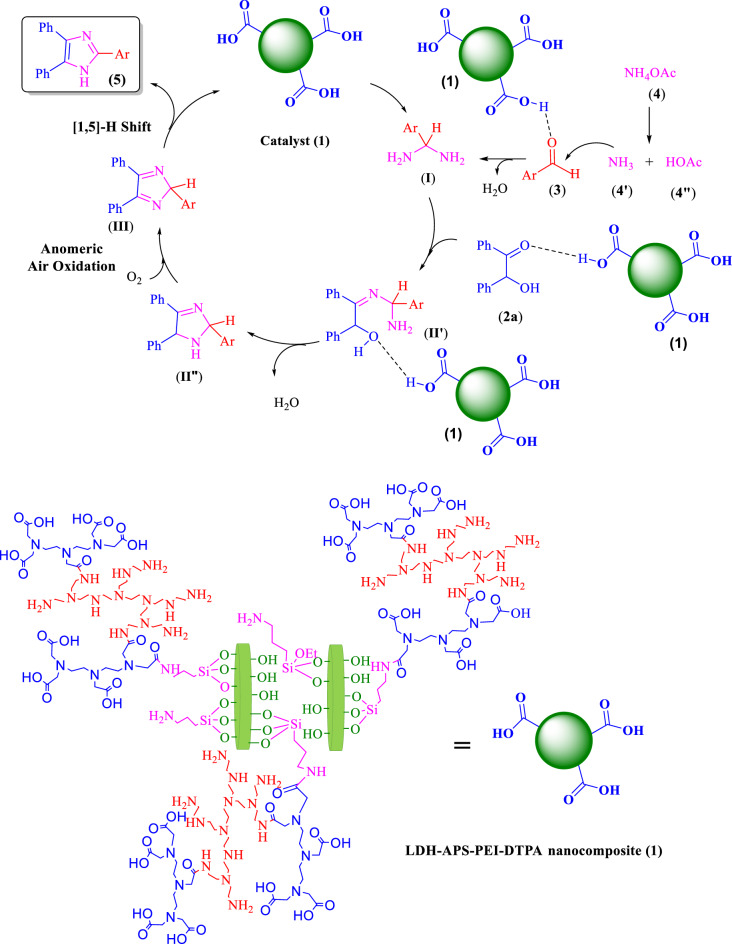
The proposed mechanism for the synthesis of imidazole derivatives using benzoin in the presence of LDH–APS–PEI–DTPA nanocomposite (**1**) catalyst.

The proposed mechanism is shown in Fig. 8. As can be seen LDH–APS–PEI–DTPA nanocomposite (**1**) has mild Brønsted acidic properties. Here, the LDH–APS–PEI–DTPA nanocomposite (**1**) by acidic groups activates the carbonyl group in aldehyde derivatives (**3**) by forming a hydrogen bond to enhance the nucleophilic addition of ammonium acetate (**4**) to form an intermediate aminal (**I**). Also, LDH–APS–PEI–DTPA nanocomposite (**1**) by acidic groups activates the carbonyl group in benzoin (**2**) then intermediate (**I**) reacts with benzoin (**2**) to form intermediate (**II′**) and The water molecule is removed. In the following, oxidation of intermediate (**II″**) in the presence of air is facilitated by the anomeric effect of the adjacent N atoms of the benzoin C-H bond to form the cyclic intermediate (**III**). Then, [1,5]-H shift of the intermediate (**III**) results in imidazole **5** derivative as the final product. On the other hand, the by-products in these reactions are two or three H_2_O molecules. This confirms that the method is environmentally friendly^52^.

**Figure 9:**
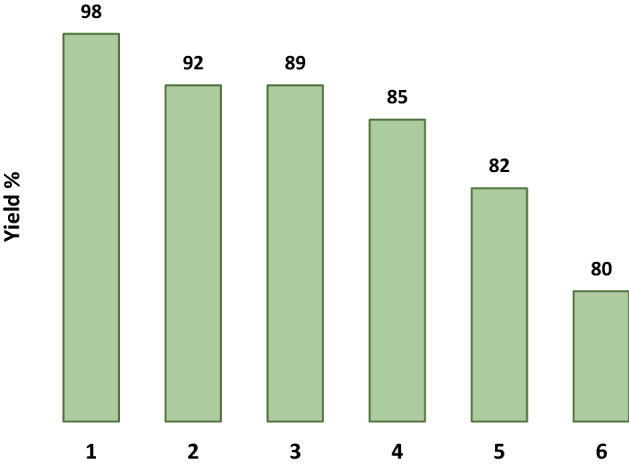
Reusability of the LDH–APS–PEI–DTPA nanocomposite (**1**) in the reaction with **5a**.

One of the interesting advantages of LDH–APS–PEI–DTPA nanocomposite (**1**) is its recyclability and reusability in subsequent reactions. In order to evaluate the reusability of the catalyst, the LDH–APS–PEI–DTPA nanocomposite (**1**) was collected after filtration washed with distilled water and ethanol and then dried at 70 °C. The recycled catalyst was then reused in the model reaction. This process was repeated five times with no significant reduction in the catalytic efficiency of LDH–APS–PEI–DTPA nanocomposite (**1**, Fig. 9).

To demonstrate the performance of the LDH–APS–PEI–DTPA nanocomposite (**1**) as catalyst, a comparison was made with the previously reported catalysts for the synthesis of imidazole derivatives. As shown in Table 3, the synthesis of imidazole derivatives in the presence of LDH–APS–PEI–DTPA nanocomposite (**1**) has some advantages over other reported catalysts such as lower catalyst loading, shorter reaction time, and environmentally friendly reaction conditions. Therefore, the composite prepared here is a more efficient catalyst for the preparation of imidazole derivatives with high-efficiency and shorter time than the previously reported catalysts.

**Table 3:** Comparing the catalytic performance of the LDH–APS–PEI–DTPA nanocomposite (**1**) with the previously reported catalysts for the synthesis of imidazole derivatives.

Entry	Catalyst	Conditions and catalyst loading	Time (min)	Yield (%)	Ref.
1	CSC-Star	EtOH, reflux, 20 mg	180	93	^53^
2	Mo-salen complex nanoparticles onto silica	EtOH, 50 °C, 2.5 mol%	90	90	^54^
3	Ferric(III) nitrate supported on kieselguhr	Solvent-free, 120 °C, 160 mol%	60	89	^55^
4	L-Proline	MeOH, 60 °C, 15 mol%	540	88	^56^
5	LDH–APS–PEI–DTPA nanocomposite (1)	EtOH, reflux, 5 mg	45	95	This work

## Experimental section

### Reagents and apparatus

The chemicals were purchased from Aldrich or Merck with high purity and used in experiments without purification. LDH–APS–PEI–DTPA nanocomposite using analysis FTIR (Shimadzu 8400s), EDX (Numerix DXP-X10P), XRD patterns (TW 1800 diffractometer with CuKa radiation (λ = 1.54050 Å)), FESEM (TESCAN-MIRA3), BET (ASAP 2020 micromeritics) and TGA (Bahr Company STA 504) were examined.

### Preparation of Mg–Al LDH

Mg Al-layered double hydroxide (Mg Al-LDH) was prepared via the urea-assisted co-precipitation method^14^. cetyltrimethylammonium bromide (CTAB, 2 g) was dissolved in the aqueous solution of urea (3 M, 100 mL) at 100 °C. Then, Mg(NO_3_)_2_⋅6H_2_O (5.13 g) and Al(NO_3_)_3_.9H_2_O (3.75 g) were added to the aqueous solution. Then, mixture was stirred at 100 °C for 12 h, and then kept aging at 94 °C for another 12 h. The Mg Al-LDH suspension was centrifuged and washed with deionized water, and then dried at 90 °C for 6 h. After that, the obtained product was calcined at 650 °C for 6 h.

### Preparation of Mg–Al LDH–APS

Mg–Al LDH (1 g) was dispersed in dry toluene (20 mL). Then, (3-aminopropyl) triethoxysilane (APS, 3 mL) was added dropwise and the mixture was stirred under reflux conditions for 24 h. Finally, the precipitate was separated by filtration, washed with toluene and ethanol, and dried at 80 °C.

### General procedure for the preparation of polymer

A mixture of diethylenetriaminepentaacetic acid (DTPA, 2 g), hydroxybenzotriazole (HOBT, 1.35 g) and 1-Ethyl-3-(3-dimethylaminopropyl) carbodiimide (EDCI, 1.56 g) were dissolved in acetonitrile (10 mL). The mixture was then stirred for 1 h at room temperature. Then, polyethylenimine (PEI, 1 g) and triethylamine (0.5 mL) were added to the mixture and stirred for 12 h at room temperature. Finally, the precipitate was filtered, washed with acetonitrile and EtOH, and dried at 80 °C for 6 h.

### Preparation of LDH–APS–PEI–DTPA nanocomposite (**1**)

**Figure 10:**
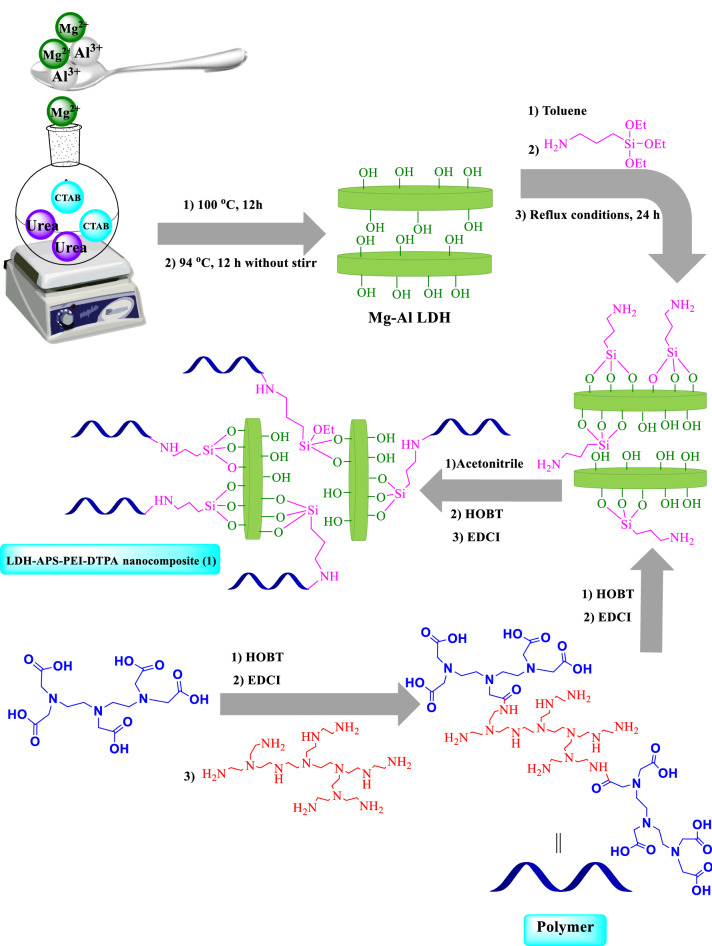
Schematic preparation of LDH–APS–PEI–DTPA nanocomposite (**1**).

Mg–Al LDH–APS (1 g) was dissolved in acetonitrile (20 mL), to which HOBT (0.6 g) and EDCI (0.78 g) were added and stirred for 30 min. After that, the prepared polymer (1 g) and triethylamine (0.5 mL) were added dropwise and stirred at 80 °C for 24 h. Finally, the precipitate was separated from the mixture, washed with acetonitrile and EtOH, and dried at 80 °C for 6 h (Fig. 10).

### General procedure for the synthesis of imidazole derivatives **5a**–**k**

Mixture of aldehyde (1 mmol), benzoin (1 mmol), ammonium acetate (2.5 mmol), and 5 mg of LDH–APS–PEI–DTPA nanocomposite (**1**) in EtOH solvent (5 mL) was stirred at 80 °C. The improvement of the reaction was checked out by TLC in a mixture of hexane and EtOAc (4:1 v/v). Eventually, the LDH–APS–PEI–DTPA nanocomposite (**1**) was filtered from the reaction mixture. Also, the LDH–APS–PEI–DTPA (**1**) nanocomposite was also washed with acetone for reuse in subsequent reaction periods.

### Spectral characterization of compounds **5c** and **5f**

#### 2-(2-Chlorophenyl)-4,5-diphenyl-1*H*-imidazole (**5c**)

Mp.: 198–200 °C; IR (KBr, cm^–1^): 3445, 3062, 1603, 1503, 1450, 1318, 1208, 1126, 764, 695; ^1^H NMR (500 MHz, DMSO-d6): δ = 7.30–747 (m, 7H), 7.6 (d, 1H, J = 7.69 Hz), 7.65 (d, 4H, J = 7.54 Hz), 8.50 (d, 1H, J = 7.80 Hz), 10.29 (br, 1H) ppm; 13CNMR (100 MHz, DMSO-d6): δ = 126.47, 126.78, 127.12, 128.81, 129.10, 129.63, 129.74, 129.89, 130.54, 130.92, 131.83, 142.73 ppm.

#### 2-(4-Nitrophenyl)-4,5-diphenyl-1*H*-imidazole (**5f**)

Mp.: 261–263 °C; FTIR (KBr, cm^–1^): 3750, 3432, 1630, 1486, 1436, 1370, 1094, 970, 832, 704; ^1^HNMR (500 MHz, DMSO-d6): δ = 7.30–7.84 (m, 13H), 7.88 (s, 1H), 10.31 (br, 1H) ppm; ^13^C NMR (100 MHz, DMSO-d6): δ = 127.54, 127.51, 128.10, 128.81, 129.05, 129.53, 129.70, 129.86, 130.49, 130.89, 132.54, 142.26 ppm.

## Conclusions

In this study, a new nanocomposite with acidic properties was successfully prepared using LDH and polymer by a suitable method and characterized by different analysis methods. Also, the morphology of LDH was altered by changing the synthesis parameters. Due to its high thermal stability and high surface area (160 m^2^ g^−1^), the new LDH–APS–PEI–DTPA nanocomposite (**1**) was used as a heterogeneous and efficient catalyst in the three-component condensation reaction of aldehyde derivatives, benzoin, and ammonium acetate for the synthesis of imidazole derivatives under mild conditions. acceptable stability and reusability with a slight reduction in activity as well as easy and fast separation of products can be considered as the main advantages of the prepared LDH–APS–PEI–DTPA nanocomposite (**1**).

## Supplementary Information

Supplementary Information.

## Data Availability

All data generated or analyzed during this study are included in this published article [and its supplementary information files].
