# Supplementary material for: Design and preparation of nanoarchitectonics of LDH/polymer composite with particular morphology as catalyst for green synthesis of imidazole derivatives
Source: Sci Rep. 2022 Jul 4;12:11288. doi: 10.1038/s41598-022-15582-z (PMC9253321; doi:10.1038/s41598-022-15582-z)
Supplement: Supplementary file 1 — Supplementary Information. [file 41598_2022_15582_MOESM1_ESM.docx]

**Supporting Information**

**Design and preparation of nanoarchitectonics of LDH/polymer composite with particular morphology as catalyst for green synthesis of imidazole derivatives**

Nastaran Ghanbari ^a^, Hossein Ghafuri *^a^

*^a^Catalysts and Organic Synthesis Research Laboratory, Department of Chemistry, Iran University of Science and Technology, Tehran 16846‑13114, Iran*

** E-mail:* [*ghafuri@iust.ac.ir*](mailto:ghafuri@iust.ac.ir)

| **Page** | **Content** |
| --- | --- |
| S1 | Title page |
| S2 | Preparation of Mg-Al LDH |
| S2 | Preparation of Mg-Al LDH-APS |
| S2 | General procedure for the preparation of polymer |
| S2 | Preparation of LDH-APS-PEI-DTPA nanocomposite (**1**) |
| S4 | Characterization of the LDH-APS-PEI-DTPA nanocomposite (**1**) |
| S7 | General procedure for the synthesis of imidazole derivatives **5a-k** |
| S7 | Chemical characterization of 2-(2-Chlorophenyl)-4,5-diphenyl-1H-imidazole (**5c**) |
| S10 | Chemical characterization of 2-(4-Nitrophenyl)-4,5-diphenyl-1H-imidazole (**5f**) |

**Preparation of Mg-Al LDH**

Mg Al-layered double hydroxide (Mg Al-LDH) was prepared using the urea-assisted co-precipitation method. First, cetyltrimethylammonium bromide (CTAB, 2 g) was dissolved in the aqueous solution of urea (3 M, 100 mL) at 100 °C. Then, Mg(NO_3_)_2_⋅6H_2_O (5.13 g) and Al(NO_3_)_3_.9H_2_O (3.75 g) were added to the aqueous solution. The mixture was transferred to a glass flask (200 mL) equipped with a reflux condenser, and stirred at 100 °C for 12 h, and then kept at 94 °C for another 12 h without stirring. The MgAl-LDH suspension was centrifuged and washed with deionized water until reaching pH 7, and then dried at 90 °C for 12 h. After that, the obtained product was calcined at 650 °C for 6 h.

**Preparation of Mg-Al LDH-APS**

Mg-Al LDH (1 g) was dispersed in dry toluene (20 mL). Then, (3-aminopropyl) triethoxysilane (APS, 3 mL) was added dropwise and the mixture was stirred under reflux conditions for 24 h. Finally, the precipitate was separated by filtration, washed with toluene and ethanol, and dried at 80 °C.

**General procedure for the preparation of polymer**

A mixture of diethylenetriaminepentaacetic acid (DTPA, 2 g), hydroxybenzotriazole (HOBT, 1.35 g) and 1-Ethyl-3-(3-dimethylaminopropyl) carbodiimide (EDCI, 1.56 g) were dissolved in acetonitrile (10 mL). The mixture was then stirred for 1 h at room temperature. Then, polyethylenimine (PEI, 1 g) and triethylamine (0.5 mL) were added to the mixture and stirred for 12 h at room temperature. Finally, the precipitate was filtered, washed with acetonitrile and EtOH, and dried at 80 °C for 6 h.

**Preparation of LDH-APS-PEI-DTPA nanocomposite (1)**

Mg-Al LDH-APS (1 g) was dissolved in acetonitrile (20 mL), to which HOBT (0.6 g) and EDCI (0.78 g) were added and stirred for 30 min. After that, the prepared polymer (1 g) and triethylamine (0.5 mL) were added dropwise and stirred at 80 °C for 24 h. Finally, the precipitate was separated from the mixture, washed with acetonitrile and EtOH, and dried at 80 °C for 6 h (**Figure. 1**).

**Fig. S1.**Schematic preparation of LDH-APS-PEI-DTPA nanocomposite (**1**).


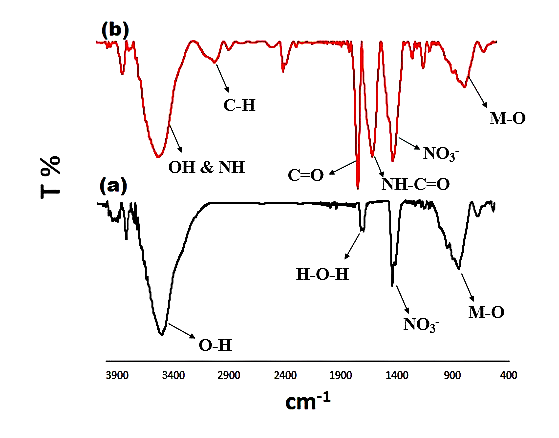


**Fig. S2.** FTIR spectra of Mg-Al LDH (**a**) and LDH-APS-PEI-DTPA nanocomposite (**b**).


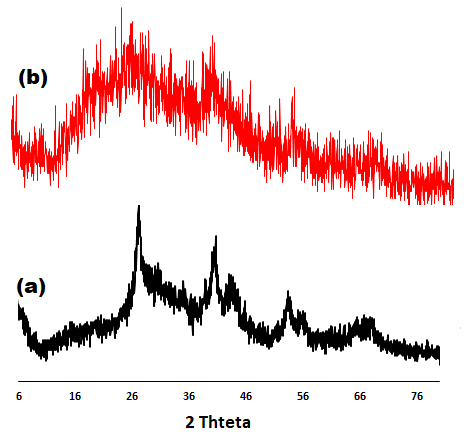


**Fig. S3.** XRD patterns of Mg-Al LDH (**a**) and LDH-APS-PEI-DTPA nanocomposite (**b**).

| 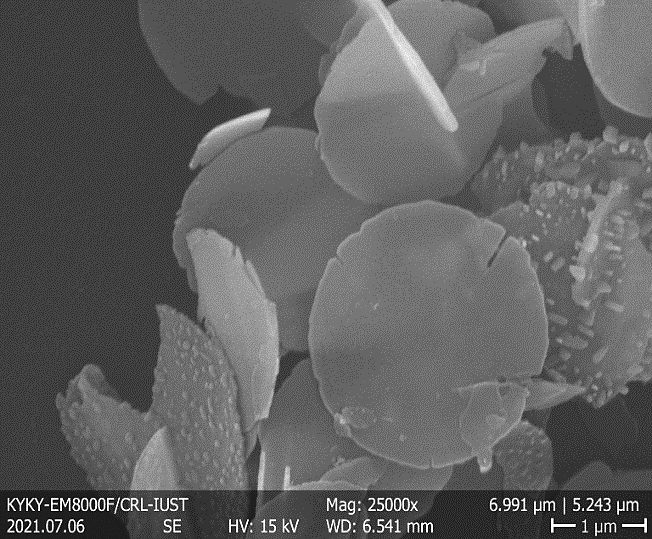  **(a)** | 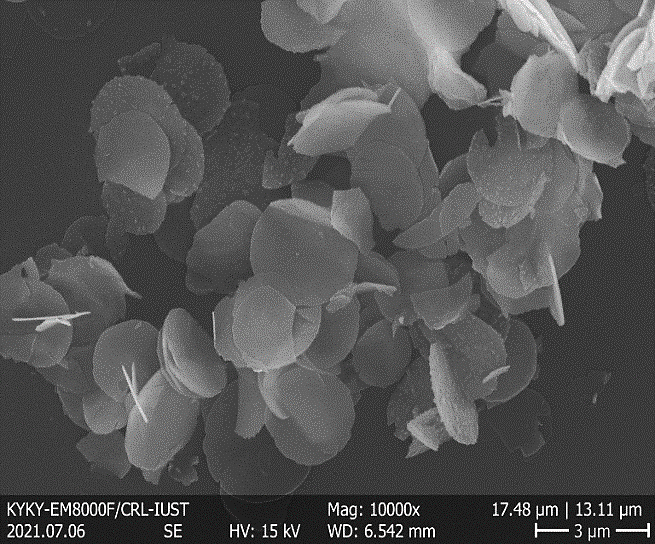  **(b)** |
| --- | --- |
| 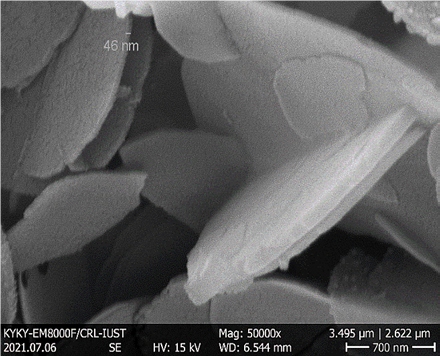  **(c)** | 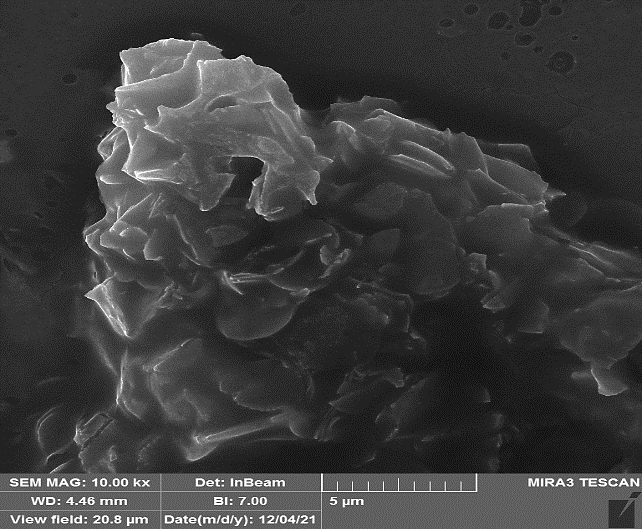  **(d)** |
| 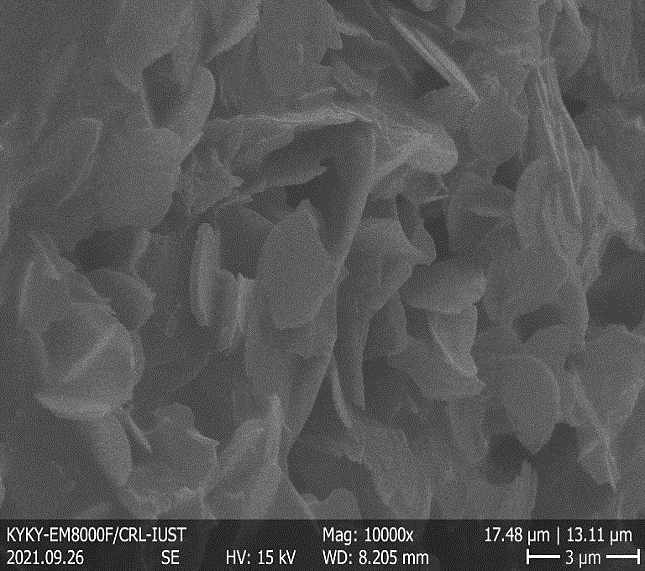  **(e)** | 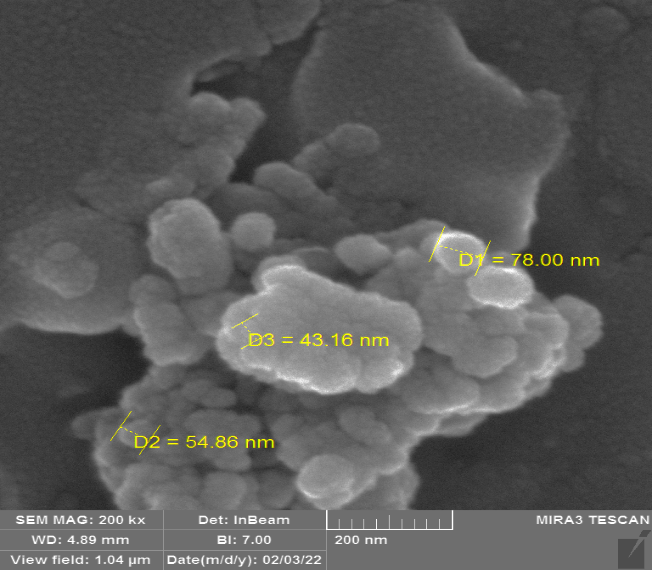  **(f)** |

**Fig. S4**. FESEM images of the Mg-Al LDH (**a, b, c**), and LDH-APS-PEI-DTPA nanocomposite (**1, d, e, f**).


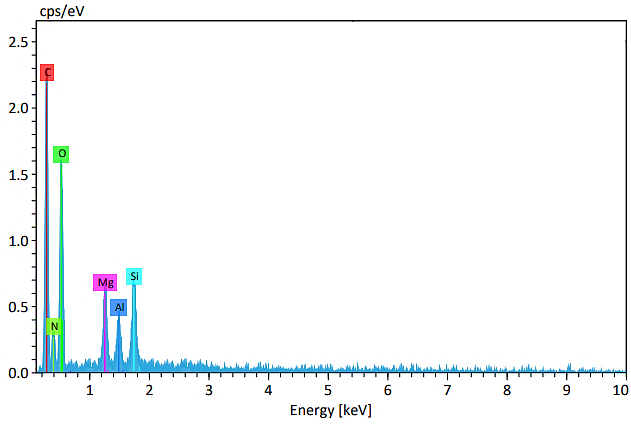


**Fig. S5.** EDX spectra of LDH-APS-PEI-DTPA nanocomposite (**1**).


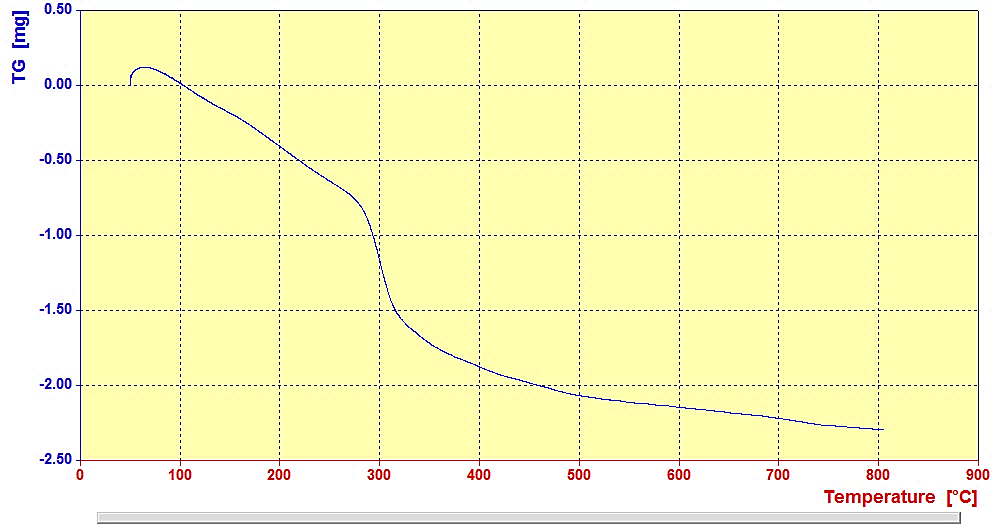


**Fig. S6.**TGA and DTA curves of the LDH-APS-PEI-DTPA nanocomposite (**1**).

**
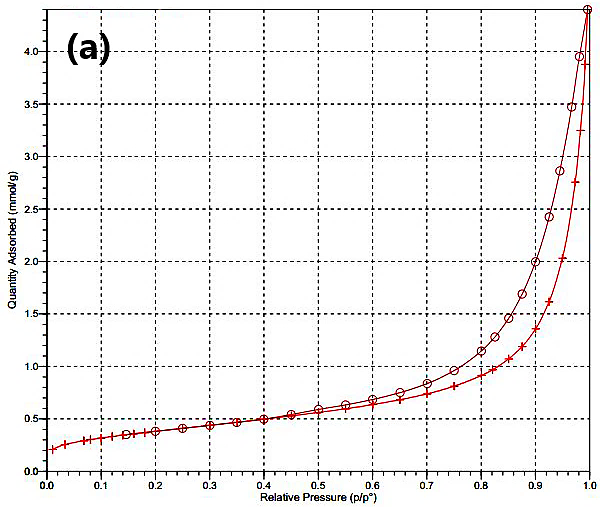
**

**
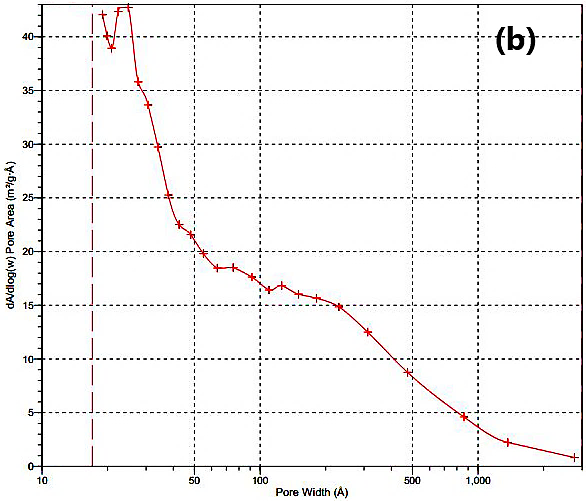
**

**Fig. S7.** N_2_ adsorption-desorption curves for the LDH-APS-PEI-DTPA nanocomposite (**1**).

**General procedure for the synthesis of imidazole derivatives 5a-k**

In a 5 mL flask, a mixture of benzoin (**2**, 1 mmol), aldehyde (**3a-k**, 1 mmol), ammonium acetate (**4**, 2.5 mmol), and 5 mg of LDH-APS-PEI-DTPA nanocomposite (**1**) in EtOH solvent (5 mL) was stirred under reflux conditions. The progress of the reaction was monitored by TLC in a mixture of hexane and EtOAc (4:1 v/v). After completion of the reaction, the LDH-APS-PEI-DTPA nanocomposite (**1**) was separated by filtration from the reaction mixture. Then, the product was recrystallized for further purifying. Also, LDH-APS-PEI-DTPA nanocomposite (**1**) was washed with acetone before using in the next runs.

**Spectral characterization of compounds 5c and 5f**

2-(2-Chlorophenyl)-4,5-diphenyl-1H-imidazole (**5c**)

Mp.: 198–200 °C; IR (KBr, cm^–1^): 3445, 3062, 1603, 1503, 1450, 1318, 1208, 1126, 764, 695; ^1^H NMR (500 MHz, DMSO-d6): δ = 7.30-747 (m, 7H), 7.6 (d, 1H, J = 7.69 Hz), 7.65 (d, 4H, J = 7.54 Hz), 8.50 (d, 1H, J = 7.80 Hz), 10.29 (br, 1H) ppm; 13CNMR (100 MHz, DMSO-d6): δ =126.47, 126.78, 127.12, 128.81, 129.10, 129.63, 129.74, 129.89, 130.54, 130.92, 131.83, 142.73 ppm.


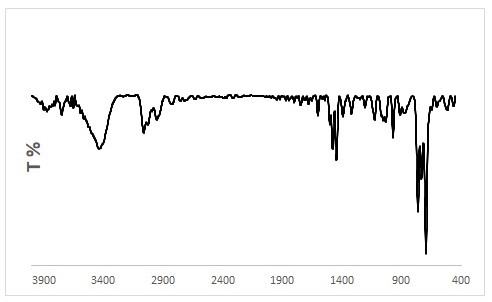


**Fig. S8.** FT-IR of 2-(2-Chlorophenyl)-4,5-diphenyl-1H-imidazole.


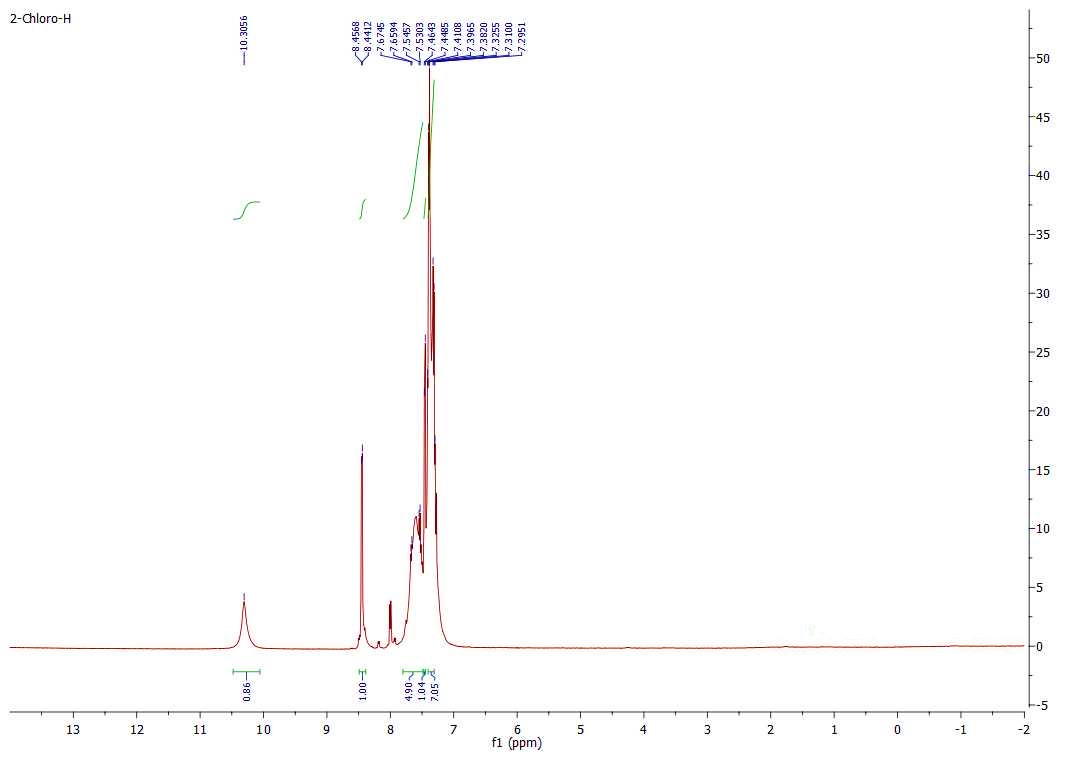


**Fig. S9.** ^1^HNMR of 2-(2-Chlorophenyl)-4,5-diphenyl-1H-imidazole.


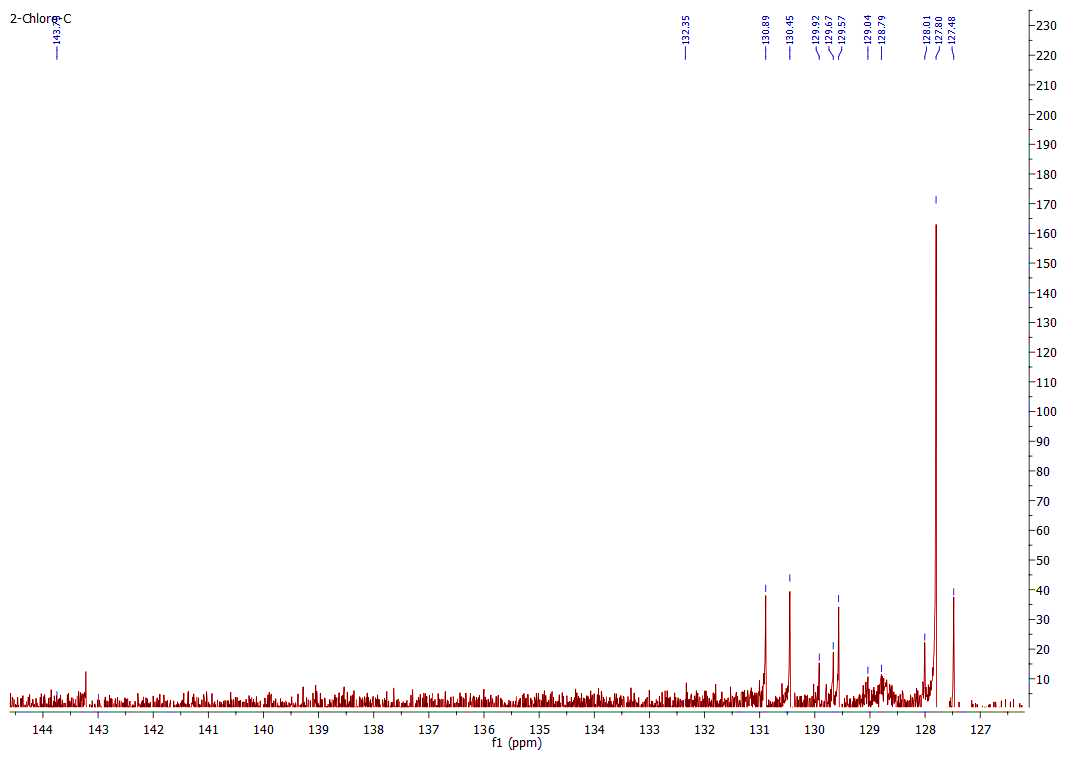


**Fig. S10.** ^13^CNMR of 2-(2-Chlorophenyl)-4,5-diphenyl-1H-imidazole.

2-(4-Nitrophenyl)-4,5-diphenyl-1H-imidazole (**5f**)

Mp.: 261–263 °C; FTIR (KBr, cm^–1^): 3750, 3432, 1630, 1486, 1436, 1370, 1094, 970, 832, 704; ^1^HNMR (500 MHz, DMSO-d6): δ=7.30–7.84 (m, 13H), 7.88 (s, 1H), 10.31 (br, 1H) ppm; ^13^C NMR (100 MHz, DMSO-d6): δ = 127.54, 127.51, 128.10, 128.81, 129.05, 129.53, 129.70, 129.86, 130.49, 130.89, 132.54, 142.26 ppm.


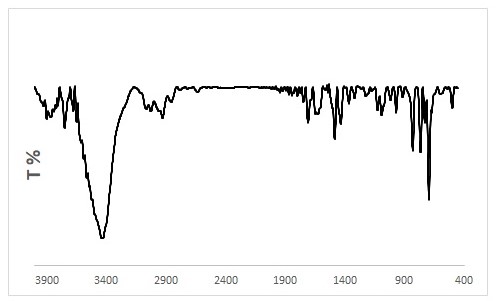


**Fig. S11.** FT-IR of 2-(4-Nitrophenyl)-4,5-diphenyl-1H-imidazole (**5f**).


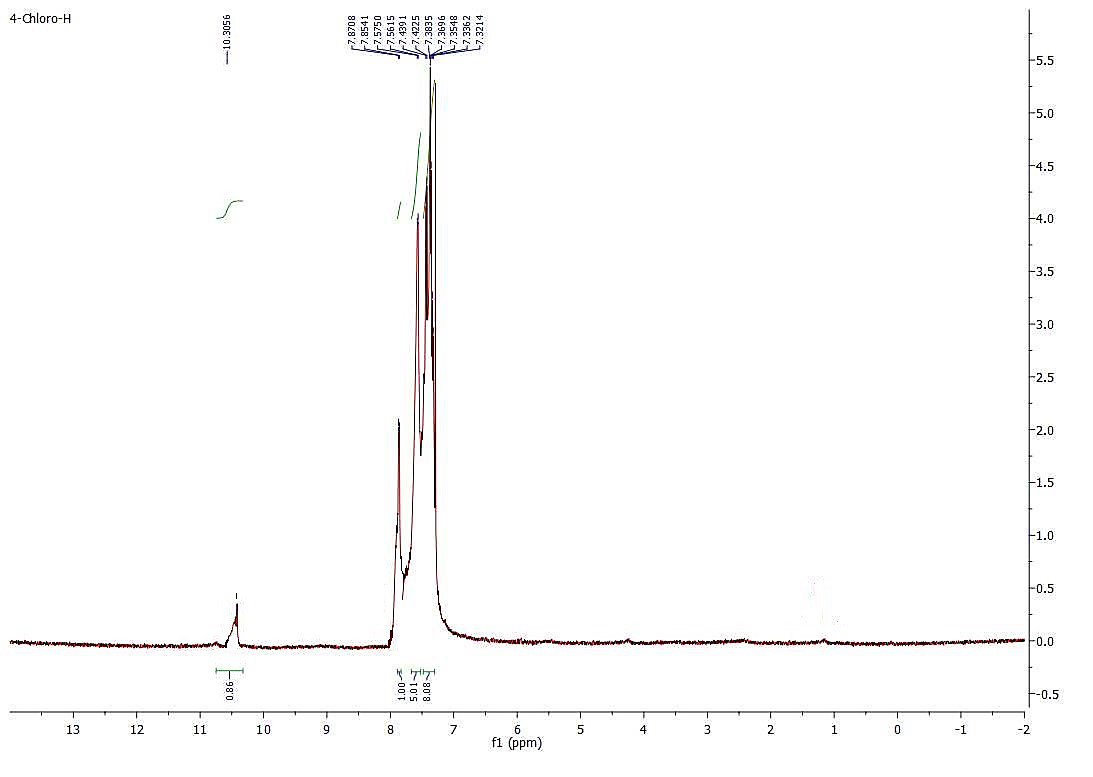


**Fig. S12.** ^1^HNMR of 2-(4-Nitrophenyl)-4,5-diphenyl-1H-imidazole (**5f**)


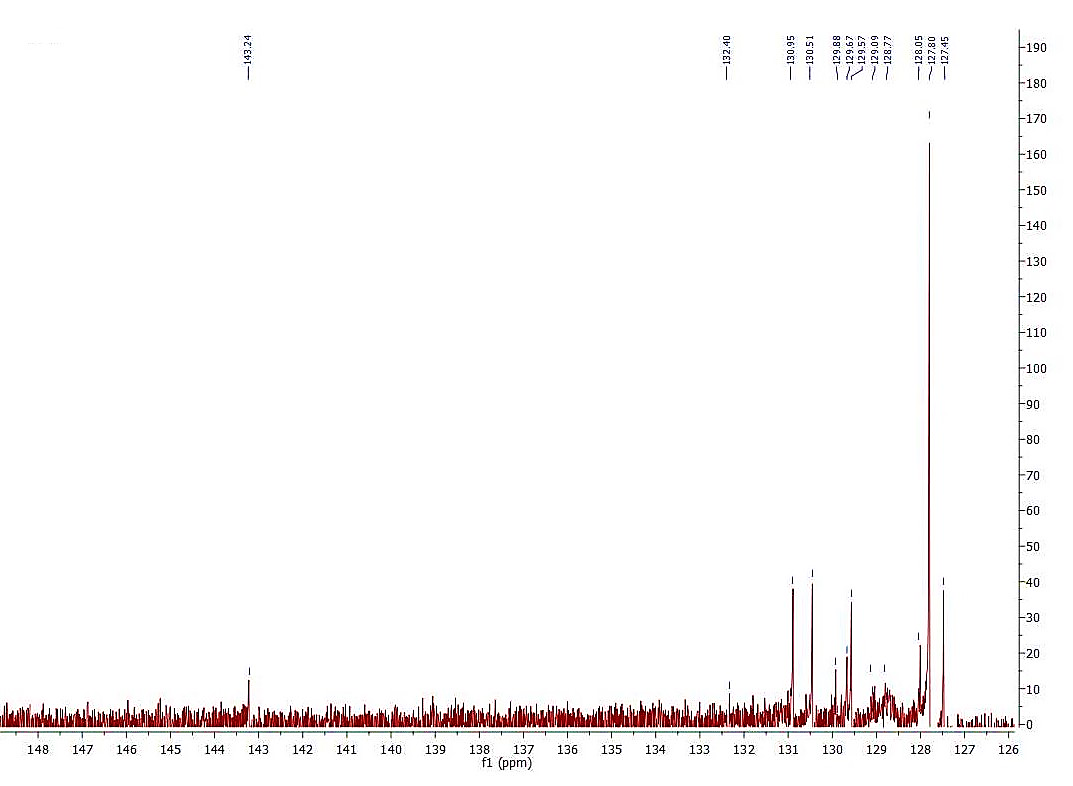


**Fig. S13.** ^13^CNMR of 2-(4-Chlorophenyl)-4,5-diphenyl-1H-imidazole.
